# The Effects of Virtual Reality Tele-exergaming on Cardiometabolic Indicators of Health Among Youth With Cerebral Palsy: Protocol for a Pilot Randomized Controlled Trial

**DOI:** 10.2196/40708

**Published:** 2022-08-17

**Authors:** Byron Lai, Drew Davis, Raven Young, Erin Kimani-Swanson, Cynthia Wozow, Huacong Wen, Yumi Kim, Jereme Wilroy, James Rimmer

**Affiliations:** 1 Division of Pediatric Rehabilitation Medicine Department of Pediatrics University of Alabama at Birmingham Birmingham, AL United States; 2 Department of Physical Medicine & Rehabilitation University of Alabama at Birmingham Birmingham, AL United States; 3 Dean's Office School of Health Professions University of Alabama at Birmingham Birmingham, AL United States

**Keywords:** disability, physical therapy, adapted physical activity, physical activity, active video gaming

## Abstract

**Background:**

Youth with cerebral palsy do not have enjoyable, accessible, and scalable exercise options that can empower them to independently maintain their cardiometabolic health.

**Objective:**

The primary aim is to examine the preliminary efficacy of a 12-week home-based virtual reality tele-exergaming intervention on several indicators of cardiometabolic health in youth with cerebral palsy compared to the wait list control. A secondary aim is to describe feasibility metrics, namely, recruitment, retention, and adherence rates; perceived enjoyment; intervention safety; and management issues. The tertiary aim is to generate a theory that reveals critical behavioral mechanisms of adherence to tele-exergaming.

**Methods:**

In this parallel group design randomized controlled trial, 34 inactive youths with cerebral palsy are randomly allocated to one of two groups: a group that immediately receives 12 weeks of virtual reality exergaming with tele–physical education or a wait list control group that undergoes their habitual activity for 12 weeks. Participants are recruited from a Children’s Hospital and community network. At baseline (week 0), week 6, and week 12, high sensitivity C-reactive protein and blood insulin, hemoglobin A_1c_, triglycerides, cholesterol, and pressure are measured by the youth and a caregiver at home using a blood spot test kit and blood pressure cuff. They will also self-measure their lung function and body weight using a peak flow meter and bathroom scale, respectively. Collections are supervised by research staff via videoconference. Changes in outcomes are compared between and within groups using exploratory statistical analyses and descriptive statistics. At postintervention or dropout, participants will undergo semistructured interviews to identify behavioral mechanisms that underly participation.

**Results:**

Recruitment procedures started in June 2022. All data are expected to be collected by October 2023. Full trial results are expected to be published by February 2024. Secondary analyses of data will be subsequently published.

**Conclusions:**

This trial tests an innovative serious exergaming virtual reality program that includes a completely remote enrollment, assessment, and intervention tele-protocol. The knowledge obtained will inform the development of a larger effectiveness trial for improving the health and well-being of youth with cerebral palsy.

**Trial Registration:**

ClinicalTrials.gov NCT05336227; https://clinicaltrials.gov/ct2/show/NCT05336227

**International Registered Report Identifier (IRRID):**

PRR1-10.2196/40708

## Introduction

Cerebral palsy is currently estimated to be prevalent among 1 million people in the United States and 23 million people worldwide [1-4]. Cerebral palsy is defined as “a group of permanent disorders of the development of movement and posture, causing activity limitations that are attributed to non-progressive disturbances that occurred in the developing fetal or infant brain” [5,6]. While cerebral palsy is not a progressive disorder, the youth age range of 13-24 years is a critical transitory stage to adopt exercise behavior that sets the course for positive health trajectories in adulthood [7]. The youth era captures both adolescents and the beginning years of early adulthood. At an early adult age, people with cerebral palsy have substantially increased risk of cardiovascular disease (CVD)–related conditions, metabolic syndrome [8], and a 3-fold increased risk of CVD mortality compared to the general population [7,9,10]. These conditions are believed to be associated with a lack of participation in regular moderate intensity exercise behavior during the adolescent period [7,11]. Adolescents with cerebral palsy engage in alarmingly low levels of exercise that far exceed those observed among peers without cerebral palsy [12-15], but they are two times more likely to exercise as early adults when exercising as adolescents [13]. Thus, there is a need to identify healthy aging strategies that support youth with cerebral palsy with interventions that reduce the risk of physical inactivity and early disease onset.

Conventional modalities of aerobic exercise such as cycling, running, and walking [16] are often not feasible or enjoyable for youth with cerebral palsy to maintain over prolonged periods of time. Approximately, 35% of youths with cerebral palsy will experience a decreased walking ability in adulthood, 9% will lose their ability to walk, and 27% will never have been able to walk [17,18]. Youth with cerebral palsy often use mobility devices such as wheelchairs, walkers, or orthotics. They are also highly susceptible to experiencing secondary conditions such as fatigue, impaired balance, and joint pain [18-21].

Despite over three decades of research, recent scoping reviews found that randomized controlled trials (RCTs) of exercise for people with cerebral palsy have low rates of participation and recruitment [22]. Of the 49 published RCTs on youth with cerebral palsy, the average sample size was 30 [22], and most of the study participants were ambulatory because wheelchair users were often excluded, which hinders the generalizability and transferability of the study findings [22-26]. Programs with on-site data collection or intervention procedures are difficult to recruit given the low density of youth with cerebral palsy at a single site in addition to the numerous barriers that youth with cerebral palsy may experience such as geographic (eg, distance to a fitness facility), environmental (eg, lack of accessible transportation, parks, and communities), or economic challenges (eg, cannot afford a fitness membership or one-on-one supervision by a therapist) [27,28]. Therefore, there is a need to identify innovative health-enhancing exercise interventions with strong scale-up potential for youth with cerebral palsy to confirm the effects of exercise in robust RCTs [22,23].

No RCT has demonstrated clinically meaningful improvements in cardiometabolic health in people with cerebral palsy [22,23]. Although one RCT by Slaman et al [29] reported statistical improvements in cholesterol from 6 months of loosely structured participation in local activity programs, the absolute change was small and several other blood parameters demonstrated no change. As acknowledged by the authors, these absences of changes were likely due to a suboptimal exercise dose that did not meet national and global guidelines to improve health: at least 150 minutes of moderate-to-vigorous intensity exercise per week [30,31]. Given that cardiometabolic indicators of health, including blood pressure (BP), lipids, and glucose tolerance, require gradual physiological adaptations following 1-3 months of exercise training at a moderate intensity [32-34], there is a need to identify programs that can maintain the interest of youth with cerebral palsy to participate in regular moderate intensity exercise.

Active video gaming with the latest virtual reality (VR) technology can be performed at a moderate intensity (ie, exergaming) with use of only the arms and trunk [35], making it an accessible method of improving health and function. Previous disability-related active video gaming studies primarily incorporated the Nintendo Wii and Xbox Kinect. These devices demonstrated great promise as health-enhancing exercise modalities and are still used within rehabilitation clinics nationwide, but they were discontinued by their manufacturers. In May 2019, one of the largest investments in VR gaming technology by Facebook (now referred to as Meta) led to a pivotal advancement in making VR gaming more ubiquitous for consumers: the development of the Oculus Quest. The Quest is the first VR headset of high visual quality (up to 120 frames per second) that does not require a plug-in connection to an expensive desktop gaming computer or game console. The relatively lower price of this stand-alone US $400 headset has transformed the market by allowing a wider range of people to have a fully immersive, enjoyable, and socially connected VR experience through a variety of cooperative and competitive fitness games. Moreover, true immersion within a virtual world and *built-in* internet capability, provided by the Quest, are critical elements of a potentially long-term VR gaming experience. Although the Quest may be a potentially scalable method of promoting moderate exercise among youth, simply providing them the device will likely not be sufficient to promote moderate exercise behavior over a 3-month period, as was concluded from a 3-month RCT using Nintendo Wii and Xbox Kinect technology with children [36]. Therefore, there is a need to supplement active video gaming with strategies that can promote long-term use.

Home-based telehealth programs that incorporated *virtual* behavioral coaching (telecoaching) are a desirable approach for promoting nonsupervised exercise behavior among people with disabilities who do not have convenient access to community programs. According to the Supportive Accountability Theory [37], programs that use telehealth technology can foster strong intervention adherence by providing objectively monitored feedback and promoting strong relationships with health professionals. The addition of behavioral coaching strategies such as goal-setting, confidence-building, setting reasonable expectations, and understanding benefits, underpinned by social cognitive theory [38], have been found to enhance the likelihood of promoting exercise behavior among people with disabilities [39]. Social cognitive theory provides a targeted approach toward promoting exercise behavior through four constructs: self-efficacy (perceived control over one’s behaviors), outcome expectations (anticipated physical, social, or self-evaluative outcomes from participation), sociostructural factors (facilitators and barriers), and goals (concrete plans and strategies) [38,40]. A critical advantage of incorporating social cognitive theory is that it has been tested extensively among various adult disability groups [37], this has resulted in replicable processes for increasing exercise behavior in telehealth programs. An exercise intervention that uses telehealth technology, active video gaming, and a minimal dose of behavioral coaching may promote sustainable exercise behavior among large groups of youth with cerebral palsy, as demonstrated in the largest exercise trial for youth with cerebral palsy (N=101) [41].

In a feasibility case study [35], youth wheelchair users independently maintained health-enhancing doses of moderate intensity exercise over a 1-month period at home using a VR exergaming protocol. Specifically, 2 youths with spina bifida achieved an average of 200 minutes of moderate intensity exercise per week across a 1-month period, which exceeded the recommended exercise guidelines for adults [30,31]. The 2 participants used a bundle of consumer-available equipment to exergame and objectively recorded and monitored their sessions at home. Equipment included a heart rate monitor, a mobile app, and the Quest headset installed with several active games. Participants received a low dose of weekly behavioral coaching. The youths attributed the amount of exercise they achieved to the enjoyment of the system and games, which was fostered by the immersive, “real” quality of the gameplay experience. A caregiver reported a substantial reduction in body composition for a participant after the intervention. Despite these findings, the study was only a month in duration, included youth with spina bifida, and did not measure objective health outcomes. No RCT has tested the effects of a serious telecoached exergaming program with the latest consumer-available VR equipment on cardiometabolic health among youth with cerebral palsy, particularly a trial that includes completely remote study procedures (ie, enrollment, data collection, and intervention).

The primary purpose of this study is to examine the preliminary efficacy of 12 weeks of home-based VR exercise training on several indicators of cardiometabolic health in youth with cerebral palsy compared to the wait list control (WC). The secondary purpose of this study is to explore feasibility metrics that will inform the design of a larger trial, namely, recruitment, retention, and adherence rates; perceived enjoyment; intervention safety; and management issues. The tertiary purpose of the study is to generate a theory that reveals critical behavioral mechanisms of adherence to tele-exergaming.

## Methods

### Study Design

This study is a pilot RCT using a 2-armed parallel group design to test the effect of a serious VR exergaming intervention on indicators of cardiometabolic health among youth with cerebral palsy compared with a WC. The project will include 34 youths with cerebral palsy, 13-24 years of age.

### Ethics Approval

The protocol and informed consent and assent forms were approved by the Institutional Review Board for Human Use of the University (IRB-300007833) on March 3, 2022. Eligible participants are given the study website to review the consent and assent forms in detail prior to enrollment. During a videoconference meeting on the baseline data collection visit, a member of the research team verbally reviews the consent and assent forms with the prospective participant and their caregiver. Informed consent and assent are signed digitally by the participant and their caregiver through a secure web application for building and managing online surveys and databases: Research Electronic Data Capture (REDCap). REDCap is programmed to send them a nonsigned copy of the document to their email once they sign the digital form. Participants who prefer to sign a physical copy will have a consent and assent form mailed to them. Participants are given access to a study website that contains the consent and assent forms that they can review at least 24 hours prior to their baseline data collection. Consent and assent documents are written in English.

### Eligibility Criteria

The study includes physically inactive youth with cerebral palsy who walk or use wheelchairs and mobility devices (Gross Motor Function Classification System [GMFCS] levels I-IV) [42,43].

The inclusion criteria were a medical diagnosis of cerebral palsy, being between the ages of 13-24 years to accommodate the World Health Organization definition of youth and the minimum age of 13 years specified by the Quest, and a physician’s clearance to participate.

The exclusion criteria were being physically active (defined as >150 minutes per week of moderate-to-vigorous intensity exercise in a typical week); a classification of GMFCS level V, which we have found to preclude the ability to use the Oculus Quest handheld controllers; complete blindness or deafness; and having contraindications to exercise based on the American College of Sports Medicine guidelines [44].

### Randomization Allocation and Other Trial Considerations

Participants are randomized into one of two groups—VR tele-exergaming or WC (n=17 per group)—with a 1:1 allocation ratio using a permuted block randomization approach. To balance the functional ability of participants between groups, the first 30 participants will be evenly stratified into VR tele-exergaming or WC based upon their GMFCS level [45,46]. The last 4 participants will intentionally not be stratified to prevent having to exclude prospective participants. GMFCS levels I-II are indicative of an ability to walk indoors and outdoors. Levels III-IV are indicative of ambulatory with the assistance of mobility devices or nonambulatory. The randomization sequence was generated by the project statistician using a computer-generated random schedule in permuted block (SAS V.9.4; SAS Institute). Only the project statistician is aware of the randomization schedule. Data are collected and entered into REDCap by a member of the research team. This outcome assessor will be blinded to group allocation (single-blinded trial design).

### Home-Based Intervention

**Figure 1:**
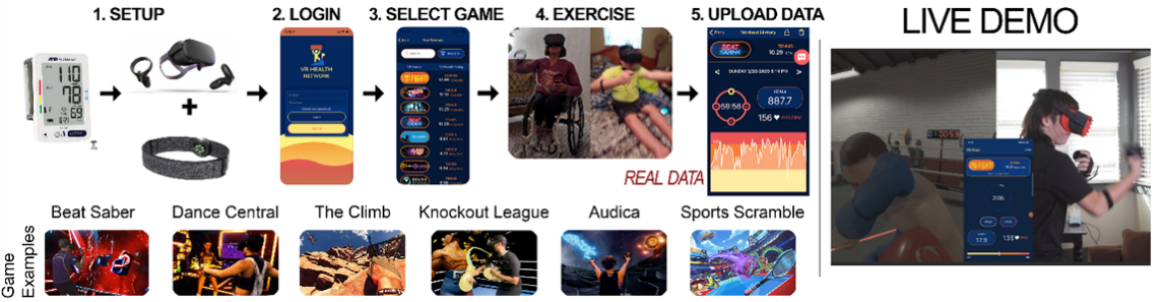
Demo of the exercise gaming protocol.

The VR intervention includes home-based exercise using the Oculus Quest, a heart rate monitor (Polar OH1), BP cuff, and mobile app (shown in Figure 1). The Quest comes with 2 handheld controllers and 9 preinstalled active games (playable while standing or sitting). The games include rhythmic movements to music and sport/recreation activities that elicit high-energy expenditure (eg, dancing, boxing, and tennis) and were chosen based on our feasibility study [35]. The Polar OH1 is worn on the forearm and is used to measure heart rate during gameplay. The OH1 has demonstrated excellent intraclass correlation coefficients (0.99) with gold standard electrocardiography [42]. Feasibility study participants reported difficulty with equipping a chest-worn monitor. During exercise, participants monitor their heart rate and exercise time via a free mobile app (VR Health) from the Google Play or Apple Store. VR Health displays exercise intensity based on an age-predicted heart rate maximum. Regarding safety, participants are instructed to refrain from exercise and call the study team if their resting heart rate is >100 bpm or BP is ≥180/110 mmHg before exercise. After exercise, the participant or caregiver uploads their exercise data from VR Health into a secure cloud server managed by study staff. Participants are instructed to exercise for 1 hour per day, with a minimum of 5 days of exercise per week. Subprescription goals are to reach 300 minutes of total play or 150 minutes per week of moderate exercise in week 1 and maintain this volume across the 12-week intervention. To enhance the likelihood that participants achieve the prescription and subgoals, participants are provided with a “Level Up” protocol, where they can earn a new game (purchased by research staff) if they achieve the prescription and at least one subgoal. The rationale for providing alternative subgoals is to accommodate the needs of participants with medications that affect their heart rate response to exercise. Intervention participants are asked to maintain their habitual diet and eating patterns throughout the 12-week period. For WC participants, they are instructed to maintain their habitual exercise, diet, and eating patterns for 12 weeks (wait period) and then receive the intervention, but no data will be collected from them during their intervention period.

### Telecoaching

The intervention includes behavioral physical education (PE) coaching through videoconference (tele-PE). Tele-PE aims to enhance adherence, provide basic exercise knowledge, monitor exercise, and increase mastery playing the games. Calls will last 15 minutes and are provided weekly in month 1, biweekly in month 2, and once at the end of month 3. Caregivers are included in the interview since caregiver knowledge and attitude are determinants of participation [43,47,48]. Tele-PE includes behavior change strategies framed within social cognitive theory [38], including planned steps toward achieving goals (earning new games via the “Level-Up” protocol), instructions on proper movement and gaming skills to increase mastery, adaptations to hardware and game settings, resolution of participation barriers, and education of the importance of exercise for health from systematic reviews. Tele-PE is delivered primarily by medical residents that are trained by the principal investigator (author BL), who has experience with adapted exercise and coaching in other telehealth trials [49-51] (NCT04264390).

### Remote Study Procedures

**Figure 2:**
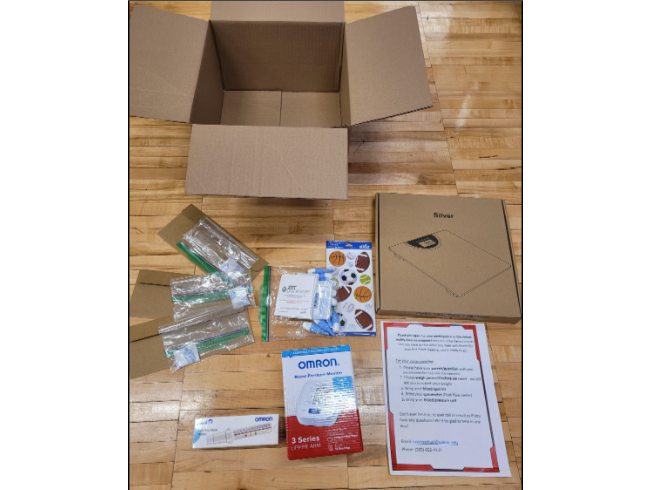
Data collection equipment package.

This trial was designed so that it could be easily replicated in a future scale-up trial. All study procedures, including screening, consent, data collection, and intervention, are conducted remotely at home. Survey data are collected through a secure web-based application for managing and creating databases (REDCap). All equipment is shipped to participants (Figure 2).

Data collection equipment includes:

- Three home dried blood spot test kits (ZRT Labs)
- Three return envelopes to ship blood samples back at the three data collection time points
- Stickers to secure the blood spot test card during collection
- BP cuff (Omron 3 Series Upper Arm, OMRON, United States)
- Peak flow meter (Peak Flow Meter PF9940, OMRON, United States)
- Low-cost bathroom scale

Outcomes for aims 1 and 2 for both the virtual reality tele-exergaming and WC groups are measured through tele-assessment at baseline (week 0), midintervention (week 6), and postintervention (week 13). The participant, a caregiver, and a research assistant meet through a Health Insurance Portability and Accountability Act–protected Zoom (Zoom Video Communications) videoconference room to complete testing synchronously. Participants are asked not to consume food or drink 10-12 hours overnight before completing a blood spot and BP test in the morning. The research assistant visually and verbally guides the caregiver through the procedures. Systolic and diastolic BP will be measured after 5 minutes of seated rest and a second time after 2 minutes of rest using a sphygmomanometer with evidence to support its accuracy and reliability for home use [52]. Lung function is measured using a low-cost, clinical, and nondigital peak flow meter. Body weight is measured using an off-the-shelf bathroom scale. For a blood spot test, capillary blood drops will be taken from the finger following a finger stick with a lancet and deposited onto a blood spot card. Blood spots are dried on the filter card for at least 2 hours before closing the cover and then dried overnight before shipping to the research team. Specimens will be stored at −70 °C until they can be shipped in bulk for testing and analysis to ZRT Laboratory, Beaverton, Oregon [53]. The blood spot test kit was designed for adult home use and has been conducted with children [54]. Participants are reimbursed with US $60 per tele-assessment session (3 × US $60 = US $180). The dollar amount is loaded onto a debit card. After completing the intervention or dropping out, the participant and a caregiver will be asked to participate in a semistructured interview through a videoconference or phone call (aim 3). Interviews will last up to 30 minutes and contain 10 general questions examining barriers and facilitators to adherence, likes and dislikes with the program and equipment, and recommendations to improve the program. General questions will include follow-up questions that probe underlying behavioral mechanisms to participation. BL is the study interviewer. He has completed over 400 interviews related to disability and exercise. Participants will be reimbursed with US $25. Interviews will be audio recorded and then transcribed for analysis. The qualitative component will be published in a secondary analysis publication.

### Outcomes

Baseline participant characteristics will include age, sex, ethnicity, physical activity level, and GMFCS level. Physical activity will be measured by the Godin Leisure-Time Exercise Questionnaire (GLTEQ) [55], and GMFCS will be obtained via parent report [45,46]. The GLTEQ is a 7-day recall, 3-item self-report questionnaire, with evidence to support its use as a valid and reliable measure of physical activity among adults with disabilities [56] and adolescents [57].

### Aim 1

Primary outcomes will include lung function, body weight, and blood tests, including high-sensitivity C-reactive protein (hsCRP); hemoglobin A_1c_ (HbA_1c_); and fasting insulin, triglycerides, and cholesterol (total, low-density lipoprotein [LDL], and high-density lipoprotein [HDL]). The ZRT Lab dried blood spot test has demonstrated excellent validity with venous serum samples (eg, hsCRP, *r*=0.99; fasting insulin, *r*=0.93; fasting triglycerides, *r*=0.95) [53,58].

#### High-Sensitivity C-Reactive Protein (mg/L)

C-reactive protein is a critical marker of inflammation that contributes to proinflammatory and prothrombotic elements of CVD risk. A single hsCRP measure is a strong predictor of myocardial infarction or coronary heart disease mortality, and several other diseases of the circulatory system in people without a history of such conditions [59]. Changes in hsCRP may occur from as early as 8 weeks of exercise [33].

#### Hemoglobin A_1c_ (mmol/mol)

HbA_1c_ is a measure of red blood cell mean hemoglobin glycation over the previous 3 months. Exercise interventions for 1 month without a dietary component can expect a small to moderate effect on HbA_1c_ from 1 month of training [34].

#### Fasting Insulin (μIU/mL)

High fasting insulin indicates the presence of insulin resistance, whether or not an individual shows glucose intolerance. Exercise interventions without a dietary component can expect a small beneficial change in fasting insulin levels from 1 month of training [34].

#### Fasting Triglycerides (mg/dL)

A triglyceride level >150 mg/dL is largely supported as an indicator of CVD risk [60,61]. Exercise interventions without a dietary component can expect a small beneficial change in triglyceride levels following 1 month of training [34] even among people with normal triglyceride levels [62].

#### Fasting Cholesterol (mg/dL)

Abnormalities in the lipid profile, including high total cholesterol, high LDL cholesterol, and low HDL cholesterol, are predictors of future CVD among young and middle-aged people [63,64]. Exercise interventions without a dietary component can expect a small effect after 1 month [34].

#### Blood Pressure (mmHg)

Elevated BP during childhood and adolescence is associated with intermediate markers and CVD-related events in adulthood [65]. Moderate intensity exercise is negatively associated with BP [11]. Small changes in BP can occur from as little as 1 month of endurance training [66].

### Aim 2

Feasibility is measured through process and management metrics [67]. Recruitment, retention, and adherence rates are recorded throughout the study and will be descriptively reported at the end of the study. Recruitment rate is described as the number of people contacted via phone call divided by the number of people enrolled in the study. Retention is defined as the average number of exergaming minutes per week completed and uploaded by participants across the intervention divided by the total number of minutes prescribed (300 minutes per week). Adherence is described as the number of data collection sessions completed divided by the total number of sessions scheduled (total of 102: 34 participants with 3 data collections per participant). At weeks 0, 6, and 13, participants complete 3 questionnaires to measure enjoyment, pain, and fatigue. Enjoyment is measured via the Physical Activity Enjoyment Scale (PACES) [68]. PACES includes 18 items that are measured on a scale from 1 to 7 and has demonstrated its usefulness as a valid measure of physical activity enjoyment among a variety of age groups [68,69]. Pain and fatigue (prevalent among youth with cerebral palsy) [18] are measured by the National Institutes of Health Neuro-QoL Pediatric Pain and Fatigue [70]. Adverse events (eg, falls or injuries) and problems are recorded and reported to the university institutional review board (IRB) as appropriate. Management issues with the remote procedures are recorded and reported.

### Analyses

#### General Rules

As a preliminary study, the study is not powered for effectiveness. Findings will inform sample size and design considerations for an effectiveness trial. A CONSORT (Consolidated Standards of Reporting Trials) flow diagram will be reported. Analyses will be performed in an intent-to-treat manner. Statistical tests will be conducted using SAS software 9.4 or greater, considering 2-sided tests with an alpha level of .05.

#### Primary Aim

Descriptive statistics and exploratory statistical procedures will be presented [71,72]. Descriptive statistics will include means/medians, SDs/IQRs, effect sizes, box and whisker plots, and 95% CIs. Statistical procedures will primarily use mixed model repeated measures analysis of covariance (ANCOVA). This will allow us to test the group, time, and group by time interaction effect simultaneously while adjusting for potential differences in blood-related health outcomes or participant characteristics at baseline. Post hoc analysis will include Tukey-Kramer multiple comparisons test. Means between groups will be compared using the 2-group *t* test; changes within groups will be compared using paired *t* tests. If the results of the ANCOVA warrant further investigation, additional analyses will be conducted.

#### Secondary Aim

For the feasibility metrics (ie, recruitment, retention, and adherence rates), no a priori criteria for acceptability will be established. Questionnaire results will be descriptively reported, and changes across time will be explored using general linear mixed models techniques, such as mixed models repeated measures analyses. An appropriate structure for the covariance matrix (eg, unstructured) will be selected for these models using the final data. Post hoc analyses will be performed using the Tukey-Kramer multiple comparisons test.

#### Tertiary Aim

The qualitative component will follow Charmaz’s constructivist grounded theory framework [73], guided by the following philosophical assumptions: critical realism ontological perspective [74] and an interpretivism epistemological perspective [75]. Data will be analyzed by authors BL and YK (who is not directly involved with the intervention). Data analysis will include three phases: (1) generation of initial codes (ie, phrases that represent lines of text) and (2) focused codes (ie, phrases that represent one or more initial codes), and (3) creation of conceptual categories (ie, higher order phrases that represent focused codes) and linkages to construct a substantive theory [73]. Further details are described elsewhere [76].

#### Power

Given the lack of previous research, we are primarily interested in examining the effect estimates of tele-exergaming on blood outcomes in aim 1, which will inform sample size determinations for an efficacy trial. Thus, the sample size determination was based on a power *estimate* calculation using a noncentral T distribution approach that is intended for pilot RCTs [77]. Specifically, a sample size of 34 will allow relatively precise estimates of the treatment effect on aim 1 study outcomes, considering a modest effect (standardized difference 0.5), 2 tails, type I error rate of 5%, 90% power [77], two parallel arms with 1:1 allocation, and a 14% dropout rate. This sample size will surpass the recommendations for pilot feasibility trials of 30 [78] and 12 per group [79]. The effect size of 0.5 was based on the RCT by Slaman et al [29], which is the only RCT with a statistically significant benefit associated with cardiometabolic health (cholesterol) in youth with cerebral palsy.

## Results

This study was approved by the university IRB in March 2022. The study was initiated in June 2022, and the first participant was enrolled on June 17, 2022. Recruitment of the last participant is anticipated in Q2 of 2023.

## Discussion

### Overview

Due to alarmingly low rates of exercise participation, youth with cerebral palsy are at substantially high risk for CVD-related conditions and CVD mortality as they age into adulthood. Regular participation in aerobic exercise is an effective nonpharmaceutical method for preventing CVD and metabolic syndrome, but effective modalities such as walking, running, and cycling are often not suitable for the large demographic of youth with cerebral palsy who have reduced mobility. The growing availability of internet access and acceptance of telehealth (due to the COVID-19 pandemic) create an unprecedented opportunity to engage large underserved groups of youth with cerebral palsy in exercise behavior. When combined with recent advances in consumer-available VR video game technology, telehealth programs have the potential to create accessible and fully immersive single and multiplayer active video gaming experiences at home. This enjoyable modality of exercise may enhance the likelihood that youth with cerebral palsy maintain regular participation over periods of time that are necessary to elicit changes in cardiometabolic health. Therefore, we hypothesize that 3 months of telemonitored VR exergaming with behavioral coaching will result in greater changes in key indicators of cardiometabolic health in youth with cerebral palsy compared with a WC group that maintains habitual activity. This study is being tested among a cohort of patients from the Children’s Hospital of Alabama, Birmingham, Alabama.

### Strengths and Limitations

In addition to the intervention, another innovative component of the study is that it incorporates remote study procedures, including tele-assessment data collections. Due to difficulties with allocating transportation and the low density of youth with cerebral palsy in one geographic area, an entirely remote study procedure creates far greater accessibility than one that requires on-site visitation. Therefore, the study has strong potential to be replicated in a scale-up effectiveness trial. Regarding limitations, the sample size determination was based on a pilot estimate; therefore, the statistical analyses may not be adequately powered. Results from this study will need to be confirmed in a larger confirmatory trial. Additionally, this study requires participants to be able to use the handheld controllers and view the screen of the head-mounted display, which may not be appropriate for some youth with cerebral palsy who have functional and visual impairments.

### Conclusions

This study is testing the latest consumer-available VR gaming technology with a serious exercise prescription and behavioral telecoaching protocol on cardiometabolic indicators of health among youth with cerebral palsy. Should the results demonstrate a potential effect on outcomes, they will need to be confirmed in an efficacy and effectiveness trial.

## Appendix

Multimedia Appendix 1 Peer-review report from the Eunice Kennedy Shriver National Institute of Child Health and Human Development Special Emphasis Panel.

## Abbreviations

ANCOVA: analysis of covariance
BP: blood pressure
CONSORT: Consolidated Standards of Reporting Trials
CVD: cardiovascular disease
GLTEQ: Godin Leisure-Time Exercise Questionnaire
GMFCS: Gross Motor Function Classification System
HbA_1c_: hemoglobin A_1c_
HDL: high-density lipoprotein
hsCRP: high-sensitivity C-reactive protein
IRB: institutional review board
LDL: low-density lipoprotein
PACES: Physical Activity Enjoyment Scale
PE: physical education
RCT: randomized controlled trial
REDCap: Research Electronic Data Capture
VR: virtual reality
WC: wait list control
