# Supplementary material for: The Effects of Virtual Reality Tele-exergaming on Cardiometabolic Indicators of Health Among Youth With Cerebral Palsy: Protocol for a Pilot Randomized Controlled Trial
Source: JMIR Res Protoc. 2022 Aug 17;11(8):e40708. doi: 10.2196/40708 (PMC9434386; doi:10.2196/40708)
Supplement: Multimedia Appendix 1 [file resprot_v11i8e40708_app1.pdf]

1R03HD107598-01 Lai, Byron

RESUME AND SUMMARY OF DISCUSSION: This new application to the NCMRR Early Career Research Award (PAR-20-042) seeks support for a pilot project that uses a theory-driven protocol for remotely delivering and objectively monitoring virtual reality (VR) exergaming through telehealth in youth with cerebral palsy (YwCP). This innovative project makes a compelling case to address a documented problem in the target population. The Principal Investigator (PI) and the team are strong, so are the Approach, environment and Letters of Support. A clear path to an R01 and a reasonable timeline are strengths as well. However, a concern was raised in that children are different and therefore needs are different, and the PI was suggested to compare gaming vs. biking or walking, etc. Secondly, the review panel cautioned that Aim 2 may not be achievable and wondered what theory Aim 2 will generate, because it seemed to the panel that the social-cognitive targets are not actually being measured; the PI was further advised to consider other validated quantitative methods as well. Thirdly, the collected data were seen as limited, because additional variables related to the intervention, such as engagement, BMI, lung function, and other metabolic measures, are not listed; the PI was also thought to be limited in delivering the intervention. Next, it appeared to the review group that the age range and the GMFCS I-IV range are too large. Other issues were noted, such as minimal efforts of Co-Is, difficulty in recruiting children, availability of technology, etc. Overall, the enthusiasm for the work was generally high, and it was rated as an excellent application that has the potential to change paradigm in the field.

DESCRIPTION (provided by applicant): Due to alarmingly low rates of exercise participation, youth with cerebral palsy (YwCP) are at substantially high risk for cardiovascular disease (CVD), related conditions, and CVD mortality as they age into adulthood. Regular participation in aerobic exercise is an effective non-pharmaceutical method for preventing cardiovascular disease and metabolic syndrome, but effective modalities such as walking, running, and cycling are often not suitable for the large demographic of YwCP who have reduced mobility. The growing availability of internet access and acceptance of telehealth (due to the coronavirus pandemic) create an unprecedented opportunity to engage large, underserved groups of YwCP in exercise behavior. When combined with recent advances in consumer-available virtual reality (VR) video game technology, telehealth programs have the potential to create accessible and fully immersive single- and multiplayer active video gaming experiences at the home. This enjoyable modality of exercise may enhance the likelihood that YwCP maintain regular participation over periods of time that are necessary to elicit changes in cardiometabolic health. This application proposes a pilot project that builds upon our recent feasibility work. The intervention will utilize a theory driven protocol for remotely delivering and objectively monitoring VR exergaming through telehealth. The procedures will be purely telehealth driven and include remote screening, consent, data collection, and intervention procedures. Participants will be recruited from a children's hospital, adult rehabilitation clinic, and network of community organizations. Thirty-four YwCP will be randomized to one of two groups: 1) 12 weeks of VR exergaming plus behavioral tele-physical education coaching (VRT) or a 2) 12-week waitlist control (WC) that undergoes habitual activity before receiving VRT. VRT participants will be prescribed to complete at least 150 min per week of moderate-intensity exercise. Using exploratory statistical procedures, this pilot study will compare changes in high sensitivity C-reactive protein and blood insulin, hemoglobin A1c, triglycerides, cholesterol and pressure between the VRT and WC only groups. At 0, 6, and 12 weeks, cardiometabolic outcomes will be measured at the home via tele-assessment. Additionally, the study will qualitatively explore behavioral mechanisms that underly participation via semi-structured interviews with participants from both groups at post-intervention or dropout. Using a Grounded Theory approach, participant feedback will be constructed into a substantive theory that can maximize long-

term engagement of YwCP in tele-exergaming. Study findings will be used to inform the development of a telehealth efficacy trial that can be easily replicated across various sites and settings.

PUBLIC HEALTH RELEVANCE: This entirely home-based, pilot study will include remote data collection and intervention procedures that will inform a larger, definitive efficacy study that can be easily implemented by researchers and health professionals across a variety of settings. The study findings will examine the potential benefits of an enjoyable virtual reality exergaming program for improving key indicators of cardiometabolic health and will strengthen our understanding of the potential benefits of eHealth to eliminate barriers to exercise participation among youth with disabilities.

CRITIQUE 1

Significance: 1
Investigator(s): 2
Innovation: 2
Approach: 2
Environment: 1

Overall Impact: The application is submitted by Dr. Lai, an Assistant Professor at the University of Alabama, and focuses on promoting exercise among youth with cerebral palsy (YwCP) aged 13-24 years. Prior research has shown these individuals to be at higher cardiometabolic risk at a young age compared with non-CP controls, purportedly due to inadequate exercise options that result in sedentary lifestyles. The application will test a pilot clinical trial of 34 inactive YwCP individuals, whom are randomized (1:1 allocation) to receive either a multifaceted intervention that includes videogaming technology through virtual reality (VR), low-dose behavioral physical education coaching, and home monitoring (intervention abbreviated “tele-PE”) for 12 weeks, versus a waitlist control group (abbreviated “WC”). Aim 1 will examine changes in cardiometabolic risk factors (CRP, insulin, A1c, lipids, blood pressure) between groups at serial time points (baseline, 6 weeks, 12 weeks). Aim 2 will obtain qualitative data from study participants to reveal behavioral mechanisms of adherence to tele-gaming. The findings will prepare for an R01 that studies tele-PE on a larger scale. The entire trial is designed to be conducted remotely at home, with equipment shipped to participants and assessments performed via videoconference. Outcomes from blood testing will be used to inform sample size for a subsequent, more definitive (R01-level) efficacy trial.
The applicants are well positioned for success given their collective expertise in YwCP and rehabilitation science, as well as the institutional support (that includes a new telehealth center) and access to potential study participants. Innovation is heightened by the novel intervention (VR videogaming), as well as study design that expands access beyond traditional trials in this population (as non-ambulatory people will still be included, and all study assessments will be conducted virtually). There is a very strong institutional commitment to the PI Dr. Lai. Minor concerns include Dr. Lai being responsible for delivering most of the Tele-PE intervention which limits scalability, as well as the requirement that study participants have electronic devices and WiFi networks at home which may limit access to more affluent populations. Nonetheless, on balance, this application has the potential to create new horizons in the area of rehabilitation research among YwCP populations and is highly recommended.

1. Significance
Strengths

- Establishes that current paradigms are ineffective; to date most exercise trials excluded participants unable to walk, had small sample sizes, and were unable to achieve the recommended 150 minutes per week of moderate-intensity exercise.
- Applicants therefore establish the need for alternative strategies that promote exercise among YwCP in order to reduce cardiometabolic risk, specifically focusing on upper extremity exercise which expands options to a larger population.
- Appropriately identify that providing people with a VR gaming console alone (without human support) is likely to fail, based on prior work.
- Straightforward, understandable conceptual model (Figure 1). Applicants postulate that behavioral coaching will promote sustained exercise, which is grounded in Social Cognitive Theory (which includes self-efficacy, outcome expectations, sociostructural factors, and goals).
- Applicants make a clear case that this grant will obtain pilot data for effect estimates that will inform a subsequent R01 application.

2. Investigator(s)
Strengths
- PI (Dr. Lai) is an Assistant Professor with a PhD in rehabilitation science and who has completed two postdoctoral fellowships in telerehabilitation (adult and pediatric) and has achieved early grant support as PI or Co-I on several projects (NIH and foundation).
- Co-I (Dr. Rimmer) directs two federally funded rehabilitation research centers and has extensive experience in this field.

Weaknesses
- Three Co-Is listed (Dr. Rimmer, Dr. Gower, Dr. Davis), and one collaborator (Dr. Powell), all at minimal effort (0.12 calendar months). Unclear whether scope of their work (e.g., assistance with recruitment, consent, data analysis) can be accomplished with such minimal effort. Understandably this is a relatively small budget grant which limits effort allocation.

3. Innovation
Strengths
- The intervention itself (VR gaming) is innovative given that it is so new.
- Investigators have built in telehealth procedures for assessment. The entire trial will be done remotely which will serve to alleviate fears over COVID-19 and improve access for people with transportation difficulties.
- The use of consumer-facing technology enables scalability for subsequent trial.

Weaknesses
- This Section is only 5 lines and would have benefitted from more in-depth description of innovative components.

4. Approach
Strengths
- Supportive preliminary data are provided from 2 individuals who received tele-PE.
- Design is inclusive of non-ambulatory YwCP.
- Feasibility of recruitment demonstrated by the following: (1) PI (Dr. Lai) has experience with recruiting YwCP participants for a separate study; (2) UAB and Spain Rehabilitation Center have 500 individuals who meet study criteria; (3) Lakeshore Foundation provides database of additional potential YwCP for recruitment.
- Laboratory testing will explicitly be used to determine effect estimates for a larger trial (R01-level application).

Weaknesses

- PI personally delivering the Tele-PE intervention is a potential barrier to scalability and reproducibility.
- Surprising that weight/BMI is not included as an outcome measure for Aim 1, given ease of obtaining and link to cardiometabolic risk.
- While conducting the trial remotely has the potential to expand access, this could also exclude individuals with economic deprivation – as they may lack technology capabilities that enable videoconferencing (e.g., home WiFi, portable electronic devices).

5. Environment
Strengths
- Co-I Dr. Rimmer (in Biosketch) states he will provide “unlimited access to our new $10M telehealth center at Lakeshore foundation to support this study.” This underscores a strong institutional environment and commitment to the PI.
- Letter of Support from Dr. Rimmer further states “As someone who has dedicated 40 years to examining novel exercise interventions for youth and adults with disabilities, I have never been as optimistic about any study design as I am about this proposal.” This is a strong endorsement of institutional support for this project.
- Strong additional Letter of Support from Dr. Davis (Division Director, Division of Pediatric Rehabilitation Medicine)
- The Applicant already has an institution-supported faculty position (with effort on other research grants) at time of application.

Study Timeline
Strengths
- Adequate to complete within designated time frame

Protections for Human Subjects: Acceptable Risks and/or Adequate Protections

Data and Safety Monitoring Plan: Acceptable

Inclusion Plans
- Sex/Gender: Distribution justified scientifically
- Race/Ethnicity: Distribution justified scientifically
- Inclusion/Exclusion Based on Age: Distribution justified scientifically

Resource Sharing Plans: Acceptable

Budget and Period of Support: Recommend as Requested

CRITIQUE 2

Significance: 2
Investigator(s): 2
Innovation: 3
Approach: 4
Environment: 2

Overall Impact: The premise of this project is based on the observation that cerebral palsy (CP) children due to motor dysfunction cannot go outside to do exercise and therefore develop cardiometabolic disease. Therefore, there is an urgent need for exercise interventions that are beneficial for maintaining cardiometabolic health in children. The exercise regimen should be age appropriate, safe, and accessible given the lack of evidence-based programs available for cerebral palsy. The PI proposes to develop an entirely home-based telehealth program with remote screening, data collection, and exergaming. The program will include the latest cost-efficient, active videogaming technology through virtual reality (VR); low-dose behavioral physical education coaching through videoconferencing (tele-PE); home-measurement techniques with strong psychometric properties; and telemonitoring equipment to objectively record and monitor exercise training at the home. The strengths include that in one randomized controlled trial of exercise demonstrated statistically significant benefits to cardiometabolic risk factors among people with cerebral palsy. However, it is not possible for CP children. Therefore, the PI is developing the appropriate sample size and programmatic considerations for a large, definitive tele-exergaming efficacy trial in YwCP (one of the most common causes of childhood physical disability). This pilot study will examine the feasibility and preliminary efficacy of 12-weeks of VR tele-exergaming on key indicators of cardiometabolic health. The PI and team are excellent and has expertise to carry out the proposed studies. The weaknesses are the lack of the supporting data on video gaming and exercise. These two methods may have two separate mechanisms. Therefore, it would be difficult to determine cause and effect of benefits. The scientific rigor lacks blind folded statistical analysis. The enthusiasm is low for this application.

1. Significance
Strengths
- The premise of this application is that there is a need of mental and physical exercise for CP children.
- There is a need for prevention of cardiometabolic problems in CP children.

Weaknesses
- It would be surprising if only tele-virtual gamming is sufficient to mitigate cardiometabolic complication in CP children.

2. Investigator(s)
Strengths
- The PI, Dr. Byron Lai, PhD, Principal Investigator is an Assistant Professor in the Division of Pediatric Rehabilitation Medicine, Department of Pediatrics at the University of Alabama at Birmingham (UAB).
- Dr. Lai is also a faculty member within the UAB/Lakeshore Research Collaborative and serves as a key scientist within the Information and Communication Technology (ICT) core. In this role, he advises the development and oversees the implementation of telehealth projects
- The other investigators, Drs. James Rimmer, PhD, Barbara Gower, PhD, Drew Davis, MD, Danielle Powell, MD, are excellent.

Weaknesses
- There is no expert collaborator on exercise regimen.

3. Innovation
Strengths
- The idea, that there is a need for tele-virtual gamming type of exercise for CP children, is novel.
- The programmatic considerations for a large, definitive tele-exergaming efficacy trial in YwCP, is innovative.

Weaknesses
- There is no innovation in the technology.

4. Approach
Strengths
- Aim 1 will examine the preliminary efficacy of 12 weeks of home-based VR exercise training on cardiometabolic health in YwCP compared to wait-list control.
- Aim 2 will examine and will generate a theory that reveals critical behavioral mechanisms of adherence to tele-exergaming.
- VRT group will achieve greater changes in cardiometabolic outcomes than the WC group across a 12-week period.
- At baseline (week 0), week 6, and week 12, high-sensitivity C-reactive protein and blood insulin, hemoglobin A1c, triglycerides, cholesterol and blood pressure will be measured by the youth and caregiver at home using a blood spot test kit and blood pressure cuff.
- Collection will be supervised by research staff via videoconference.
- The scientific rigor included all age male and female children.

Weaknesses
- The approach may not distinguish the effect of tele-virtual and exercise effects on cardiometabolic effects.
- The effect of blood pressure versus metabolic changes will not be separated.
- The lung function is not measured.
- The medication these children are on may interfere the outcome of the results.

5. Environment
Strengths
- The scientific environment at Division of Pediatric Rehabilitation Medicine, Department of Pediatrics at the University of Alabama at Birmingham (UAB) and UAB/Lakeshore Research group, where work will be done, contributes excellently to the probability of success.
- The PI has institutional support, equipment and other physical resources available to the investigators adequate for the project proposed.
- The project benefits from unique features of the scientific environment(s), subject populations, or collaborative arrangements.

Study Timeline
Strengths
- Acceptable

Protections for Human Subjects: Acceptable Risks and/or Adequate Protections

Data and Safety Monitoring Plan: Acceptable

Inclusion Plans
- Sex/Gender: Distribution justified scientifically
- Race/Ethnicity: Distribution justified scientifically
- Inclusion/Exclusion of Children under 18: Including ages <18; justified scientifically

Biohazards: Acceptable

Select Agents: Acceptable

Resource Sharing Plans: Acceptable

Authentication of Key Biological and/or Chemical Resources: Acceptable

Budget and Period of Support: Recommend as Requested

CRITIQUE 3

Significance: 1
Investigator(s): 2
Innovation: 1
Approach: 4
Environment: 2

Overall Impact: This study aims to examine blood biomarkers of health resulting from a home-based exercise program using a virtual reality device in children and young adults with cerebral palsy. There is a tremendous need to improve the amount of exercise that people with CP engage in, as most people with CP do not get appropriate amounts of exercise. Lack of exercise leads to lifelong secondary health issues. This study is innovative in that it uses tele-exercise, which is particularly useful for people who cannot participate in local exercise programs/gyms. While the study is important and innovative, it would be strengthened by quantification of other important variables such as enjoyment and heart rate changes. Clarity is needed regarding what information will be collected on REDCap, and how this information will inform future studies.

1. Significance
Strengths

- Youth with CP often do not get sufficient levels of exercise, which can lead to long-term declines in cardiovascular health, muscle strength, and more.
- A virtual gaming system enables children to exercise at home, which is advantageous to families who do not live near an adaptive gym (and adaptive gyms are rare).
- A key to a feasible exercise program is durability of enjoyment, so that participants maintain levels of aerobic activity over a lifetime. Pilot data demonstrate that the proposed exercise activities are enjoyable over a 12-week usage.
- Little is known about how blood biomarkers might change in response to exercise in children with CP, and this study will examine several important blood markers.

2. Investigator(s)
Strengths

- Excellent training and experience with exercise and tele-rehabilitation for people with disabilities.
- Good number of first-author papers
- Letters of Support convey PI’s passion and expertise for this work.
- Strong study team/collaborators

Weaknesses

- (minor) No senior-author publications

3. Innovation
Strengths

- Youth with CP, particularly youth who require mobility devices for movement, often do not get sufficient exercise, which leads to long -erm poor health.

- The use of virtual reality is innovative.
- The ability for participants to play games with each other is exciting and will likely boost compliance and enjoyment.
- Analysis of blood samples is innovative.
- Devices are affordable, which will be useful for a larger RCT and deployment into homes as clinical practice.

4. Approach
Strengths

- The study team has successfully piloted this methodology in two kids.
- Use of Polar OH1 is scientifically validated.
- Safety of exercise is high since children will be seated.
- Enjoyment is high in the pilot work.
- Use of blood spot collection from home is feasible and correlates well with venipuncture blood analyses.
- Interviews are interesting, though unclear how that knowledge will be used in the future.
- The blood marker data will be excellent pilot data for an R01, though a broader scope of outcomes (below) would further strengthen an R01.

Weaknesses

- While I think the blood markers are interesting and important, I would like to see more robust measures of enjoyment, heart rate response, and feasibility (number of hours exercised) as primary measures.
- Will the Polar OH1 data be analyzed? The Polar OH1 could be a rich dataset, yet analysis of Polar data is not mentioned. Does the VR Health app collect Polar data, or do the participants also need to use a Polar app (Polar Beat) to send data to your lab? Or is Polar only used for safety measures? You would be missing a rich dataset if you do not make it part of the study.
- Consider adding a measure of enjoyment, such as the Physical Activity Enjoyment Scale.
- For safety outcomes, the plan is for participants to call PI if HR and BP exceed safety levels. Will BP and resting HR be documented every exercise session?
- Clarity is missing regarding REDCap surveys – what specifically will be asked, how often, and who will complete surveys? Participants, caregivers, both?
- What metrics will be captured for the delayed start group during the waiting period before they begin the exercise?
- Safety of the exercise itself – are there concerns and plans regarding the possibility of a participant hurting themselves or falling out of their chair?
- What is the specific plan for participants engaging in gaming together? Will they be paired during randomization? The amount of group interaction may impact outcomes and should be documented.
- After the 12 weeks, will the participants be allowed to keep the VR device to continue exercise?
- Budget should include some buffer for broken or lost items sent to participants.

5. Environment
Strengths

- Excellent facilities that have sufficient space for study completion (except for the freezer, which will be purchased).
- Large pool of potential participants

Weaknesses

- The application would be strengthened by description of scientific community resources available.

THE FOLLOWING SECTIONS WERE PREPARED BY THE SCIENTIFIC REVIEW OFFICER TO SUMMARIZE THE OUTCOME OF DISCUSSIONS OF THE REVIEW COMMITTEE, OR REVIEWERS' WRITTEN CRITIQUES, ON THE FOLLOWING ISSUES:

PROTECTION OF HUMAN SUBJECTS: ACCEPTABLE

INCLUSION OF WOMEN PLAN: ACCEPTABLE

INCLUSION OF MINORITIES PLAN: ACCEPTABLE

INCLUSION ACROSS THE LIFESPAN: ACCEPTABLE

COMMITTEE BUDGET RECOMMENDATIONS: The budget was recommended as requested.

Footnotes for 1 R03 HD107598-01; PI Name: Lai, Byron

Ad hoc or special section application percentiled against "Total CSR" base.

NIH has modified its policy regarding the receipt of resubmissions (amended applications).See Guide Notice NOT-OD-18-197 at https://grants.nih.gov/grants/guide/notice-files/NOT-OD-18-197.html. The impact/priority score is calculated after discussion of an application by averaging the overall scores (1-9) given by all voting reviewers on the committee and multiplying by 10. The criterion scores are submitted prior to the meeting by the individual reviewers assigned to an application, and are not discussed specifically at the review meeting or calculated into the overall impact score. Some applications also receive a percentile ranking. For details on the review process, see http://grants.nih.gov/grants/peer_review_process.htm#scoring.
